# Progress in Pediatrics

**Published:** 1902-10-18

**Authors:** 


(*To be continued.*)

### Progress in Pediatrics.

(*Continued from page* 35.)

**Acute Œdema of the Eyelids in Children.**—This peculiar symptom is discussed by M. Ferron [8] in a Paris thesis. A child goes to bed apparently in a perfectly healthy condition and awakes in the morning with one or both eyes swollen and œdematous. There is no pain, but often the child is unable to open the eye or eyes. No ocular changes are present. After from 24 to 48 hours the swelling disappears. It is a form of benign œdema which alarms by the suddenness of its appearance and surprises by the suddenness of its disappearance. Recurrences are common and run a similar course. A predisposing factor is seen in a hereditary tendency to rheumatism, arthritism and neuropathy. On physical examination one can find no renal or cardiac trouble or other possible organic basis. The prognosis is good, and no special treatment save rest is called for. A case of this nature was reported last year by Dr. Theodore Fisher,[9] the exciting cause being apparently a bath. The child was nine years old, and occasionally about half an hour after a warm bath the upper and lower eyelids swelled to the size of half a walnut. There was slight injection of the conjunctivæ, but no other symptom. The urinary and circulating systems were free from disease, and apart from the ocular condition the child appeared to be in good health. The swelling usually lasted for 12 or 24 hours.

**Venous Dystrophy.**—Dr. E. Fournier [10] holds that congenital syphilis is the cause of congenital venous dystrophy, a condition manifested by dilatation and varicosity of the superficial veins. This dystrophy occurs only in infants and is observed in the temporal and facial veins. There may be a congenital feebleness of the vascular walls and an insufficiency of the venous valves. If these dilated veins are present on the head of a newly born child, syphilis is probably the cause, and other signs of that affection will be present. The paper shows some striking illustrations of the condition described.

**Raw Meat in Pulmonary Tuberculosis.**—The value of raw meat and muscle serum, advocated by Richet and Hericourt, has been tested by Drs. Josias and Roux [11] in sixteen cases of pulmonary tnberculosis. Each of the children received daily the expressed juice of 500 grammes of raw meat; cooked meat was banished from their diet and an equal quantity of raw meat was substituted. Their conclusions are that the treatment is very valuable as long as the lesion is a purely tuberculous one; and that all active signs of disease disappear during its employment. But when other infections occur in the lungs, from outside organisms, and the blood is charged with toxins produced by these organisms, the treatment has no longer any therapeutic value in the large majority of cases.

**Spasmus Nutans.**—Dr. C. Herman [12] has recorded a case of this affection in a child who had developed the condition after an attack of measles at the age of 13 months. There was no history of traumatism, and the child lived in a rather dark room, but no artificial light was used. He refers specially to this point, as exclusion from sunlight has been assigned by some writers as the cause of nystagmus and spasmus nutans in infants. The head movements could be checked by covering the eyes. Nystagmus was also present, and could be made more prominent by fixing the head. These two conditions had lasted for four and a half months, and in addition abundant evidences of rickets were present. He thought the ultimate prognosis was good. Bromides were useless but hygienic and anti-rhachitic treatment were beneficial. Discussing this case, Dr. Stokes remarked that rickets had never been present amongst some five or six cases of spasmus nutans he had met with.

**The Heart of the Child.**—In an address on this subject, Dr. D. B. Lees [13] points out that too little attention is paid to the examination of the condition of the right auricle. In the normal heart the right auricle may be detected on percussion by a dull area about the breadth of a finger extending outwards from the sternum in the fourth right intercostal space. When the right auricle is dilated the dulness may extend to two finger breadths in the fourth space, and from a half to one finger breadth in the third, and when the dilatation is excessive the dull area is increased and may even extend to the second intercostal space. When symptoms of cardiac distress are present and dilatation of the right auricle can be made out as described above, the relief of the right heart by leeches or otherwise is urgently called for. In connection with cardiac dilatation in pneumonia it must be remembered that pallor of the face and smallness of the pulse are not necessarily contra-indications to bleeding if the right auricle is found to be dilated, and that general improvement of the circulation will follow the use of leeches.

[8] These. de Paris, 1902, and Rev. Mens. des Malad. de l'Enf. 1902, April, p. 187. [9] Trans. Soc. Study Dis. of Child., 1901, Vol. I., p. 149. [10] Rev. d'Hyg. et de Med. Inf., 1902. Vol. I., No. 1. [11] Trans. Brit. Cong. on Tuberc., 1902, Vol. III., p. 317. [12] Med. Rec., Jan. 25, 1902, p. 156. [13] Lancet, Feb. 1, 1902, p. 279.

*(To be continued.)*

